# Complexity of Diarrhea-Associated Viruses in Stunted Pigs Identified by Viral Metagenomics

**DOI:** 10.1155/tbed/1974716

**Published:** 2025-06-18

**Authors:** Qingxian Li, Jianfeng Jiang, Junhui Li, Wei Zhang, Yingxue Xin, Biao He, Sun He, Changchun Tu, Yidi Guo, Wenjie Gong

**Affiliations:** ^1^State Key Laboratory for Diagnosis and Treatment of Severe Zoonotic Infectious Diseases, Key Laboratory for Zoonosis Research of the Ministry of Education, College of Veterinary Medicine, Jilin University, Changchun 130062, China; ^2^Changchun Veterinary Research Institute, Chinese Academy of Agricultural Sciences, Changchun 130122, China; ^3^TECON Biopharmaceutical Co., Ltd., Urumqi 830000, China; ^4^Jiangsu Co-innovation Center for Prevention and Control of Important Animal Infectious Diseases and Zoonoses, Yangzhou University, Yangzhou 225009, China

**Keywords:** diarrhea-associated virus, genetics, rotavirus, viromics

## Abstract

Viral diarrhea poses a severe threat to the health and growth of piglets, especially when caused by co-infection with multiple diarrhea-associated viruses. In this study anal swabs were collected from pigs older than 3 months from a farm in Gansu province, China, and subjected to viral metagenomic analysis. They had been suffering from diarrhea and their growth was significantly retarded. A total of 18 viruses were identified by high-throughput sequencing (HTS) in pooled samples from 22 stunted pigs and (separately) three healthy pigs. They included 15 diarrhea-associated RNA viruses with five porcine rotaviruses (PoRVs), porcine epidemic diarrhea virus (PEDV), a torovirus, and a sapelovirus present only in the stunted pigs. Among the identified PoRVs, PoRVBs showed a much greater genetic diversity than other PoRVs with multiple variant gene sequences identified in segments VP1 (2), VP2 (3), VP3 (4), VP4 (5), VP7 (5), NSP1 (2), NSP3 (3), NSP4 (2), and NSP5 (4), with 1–3 new genotypes being defined within each segment except NSP5. Unexpectedly, PoRVF was identified for the first time in pigs, with all gene segments exhibiting low nucleotide (56.5%−79.4%) and amino acid sequence identities (46.2%−92.0%) with previously identified avian RVF reference strains. Phylogenetic analysis showed that multiple variant strains of PAstV2 (6) and PAstV4 (13) were found in stunted pigs, and other enteric viruses were highly homologous with reference strains. Overall, the findings indicate that the stunted pigs may serve as a hotbed for the propagation of diarrhea-associated viruses and that they should be isolated and treated as early as possible.

## 1. Introduction

The pig industry is severely affected by viral diarrhea, a common condition that can spread rapidly, leading to high morbidity and mortality. Whether in large-scale pig farms or free-range homesteads, diarrheal disease has become one of the major causes of piglet mortality [1, 2]. Mild viral diarrhea can affect their growth, while in severe cases, piglets may soon become dehydrated and die [3]. Different viruses cause diarrhea in pigs with varied symptoms of disease [4], with the most prevalent being porcine epidemic diarrhea virus (PEDV), porcine delta coronavirus (PDCoV), and porcine rotavirus A (PoRVA) [5–9]. Currently, PEDV is the primary causative agent responsible for outbreaks of piglet viral diarrhea in China, especially since 2010, causing severe watery diarrhea and high mortality of infected newborn piglets. The secondary diarrheal pathogen is PoRVA, causing a relatively mild diarrhea [10]. Sapoviruses (SaV), porcine torovirus, porcine astroviruses (PAstVs), and picornaviruses (sapelovirus, teschovirus, enterovirus G) have also been reported to be detected in diarrheic pigs [7, 10–19], and some of them were probably involved in the outbreak of porcine diarrhea. For example, enterovirus G has been shown to cause mild diarrhea, fever, and reduced body weight in infected piglets, especially for the strains with genomic insertion of the porcine torovirus-derived gene encoding papain-like cysteine protease [17]. Additionally, SaV, PAstV1, and sapelovirus have been linked to mild diarrhea, the infected piglets also displayed depression and anorexia, while it remains undetermined about the pathogenicity of piglet diarrhea for other enteric viruses, such as PAstV2, PAstV4, PAstV5, porcine torovirus (PToV), and teschovirus, because these viruses were commonly detected in both diarrheic and clinical healthy pigs, and the virulence of these viruses was not clear due to the difficulty of the in vitro virus isolation [12, 15, 16, 20–24].

Rotaviruses (RVs) are widely prevalent in humans and other animals worldwide, and can cause varying degrees of diarrheal disease [25, 26]. Based on the genetic diversity of the internal capsid protein VP6, RV can be divided into nine viral species (A–D, F–J) [27–29]. Due to its segmented genome, it can readily undergo gene reassortment to produce new mutant strains [30–33]. PoRVA, PoRVB, PoRVC, and PoRVH have been shown to replicate in pigs [25, 34–37], with infections frequently causing diarrhea in piglets. Outbreaks of diarrhea caused by PoRVA, PoRVB, and PoRVC have been reported worldwide, with an increasing incidence [25, 32].

Especially in the large-scale pig industries, co-infection with multiple porcine diarrheal viruses has been frequently observed [38–40], leading to more severe clinical symptoms and posing a major challenge for the effective control of the spread of the disease. Rapid and accurate diagnosis of the major causative agent(s) is therefore essential. Viral metagenomics has proven to be a powerful tool for identifying new viruses or the complexity of causative agents in infectious diseases of unknown etiology [41]. In this study, we present the viral metagenomic profile of pooled anal samples collected from stunted pigs and from apparently healthy pigs from a farm in Gansu province, China, together with an analysis of the genetic diversity of diarrhea-associated viruses, especially the porcine RVs (PoRVs).

## 2. Materials and Methods

### 2.1. Clinical Samples

Samples were obtained in October 2022 from a fattening pig farm of Qingshan in Gansu province (GSQS), China, that maintained about 40,000 pigs consisting of approximately 10,000 nursery pigs aged 1–2 months and 30,000 fattening pigs aged 2–8 months. The nursery pigs came from three different breeding pig farms, and some were suffering from diarrhea upon arrival at the fattening farm. However, the causative agent(s) of the disease remained unknown since virus detection was not performed. Symptomatic treatment with antimicrobial or herbal drugs resulted in the recovery of about half of the diarrhetic animals, but the growth of some was significantly retarded with body weights of <10 kg at 3 months old. Such stunted pigs were retained in a pen specifically reserved for sick and disabled pigs rather than transferring them to a fattening pen. On October 4th, 2022, anal swab samples were collected from 22 stunted pigs displayed depression, anorexia, emaciate, rough hair, and half of which showed mild diarrhea, but the duration of diarrhea was not clear as well as the body temperatures upon sampling. Samples were also taken from 3 apparently healthy fattening pigs for comparison. All samples were stored at −80°C until used.

### 2.2. Viral Metagenomics

The anal swabs from the diarrheic pigs and apparently healthy pigs were separately mixed and designated GSQS-D and GSQS-H, respectively. Samples in 1.5-mL tubes were incubated with 1 mL culture medium MEM at 4°C for 2 h and centrifuged at 10,000 × *g* for 10 min. The clarified supernatants were then passed through 0.45 μm filters. For successful construction of RNA libraries for meta-transcriptomics (MTT), 200 μL of PK-15 cell lysate treated with Trizol reagent (Invitrogen, USA) were first added to the filtered supernatant (400 μL) of each anal swab sample in order to increase the RNA concentration (>500 ng), which were then subjected to RNA extraction by Trizol. Ribosomal RNA (rRNA) was first removed by Epicenter Ribo-zero rRNA Removal Kit (Epicenter, USA), and rRNA-free residue was cleaned up by ethanol precipitation. Sequencing libraries were then generated with the rRNA-depleted RNA by the NEBNext Ultra Directional RNA Library Prep Kit following the manufacturer's instructions (NEB, USA) [42, 43]. For the construction of a DNA library by multiple displacement amplification (MDA), DNA was extracted from the filtered supernatant using the DNeasy Blood & Tissue Kit (QIAGEN GmbH, Germany), and the sequencing library was constructed using the GenomiPhi V2 DNA Amplification Kit (Cytiva, UK). DNA concentrations were determined using the Qubit 1x dsDNA HS Assay Kit (Invitrogen, USA). The constructed RNA and DNA libraries were sequenced using the Illumina NovaSeq 6000 platform (Novogene, Tianjin, China), with 6G data obtained for each library; the sequencing depth is enough for screening the viruses [44, 45].

Using BWA 0.7.17, the clean data generated by high-throughput sequencing (HTS) were initially aligned with a swine genome sequence, which was then eliminated using Samtools 1.17 to filter out unaligned segments. The purified gene data were de novo assembled into contigs using Megahit 1.2.9 employing the Kmer iterative DBG method, and aligned and annotated in the nr and nt libraries of NCBI using Diamond 2.1.8 and Nucleotide–Nucleotide BLAST 2.13.0^+^. The annotation results were validated online through the Blastx module available on the NCBI database. After screening and removal of nonviral genetic sequences, the confirmed viral sequences were aligned with the acquired contigs for quantifying the number of viral reads using Bowtie2 2.4.5.

### 2.3. Detection of Diarrhea-Associated Viruses by RT-Nested PCR (RT-nPCR)

Total RNA was extracted from each of the anal swabs using the RaPure Viral RNA/DNA Kit (Megan, China) and reverse transcribed into cDNA with M-MLV (TaKaRa, China). Specific primers for the detection of diarrhea-associated viruses by PCR were designed using Primer 5 software based on the viral contig sequences obtained above (Table 1). The PCR procedure involved an initial pre-denaturation step at 95°C for 3 min, followed by 35 cycles of denaturation at 95°C for 30 s, annealing at 55°C for 30 s, and extension at 72°C for 15 s. The final extension step was prolonged by 10 min at 72°C. Finally, the amplified PCR results were identified through electrophoresis on 1% agarose gels.

### 2.4. Sequence Comparison and Phylogenetic Analysis

The DNASTAR 7.1 software package was used to conduct multiple sequence alignments for analyzing nucleotide and amino acid sequence identities between the test viral and reference sequences, as well as for the detection of deletions and insertions in gene sequences. Geneious 11.1.2 software and the NCBI ORF finder were utilized for the prediction of putative open reading frames (ORFs) within the genomic sequences. Phylogenetic analyses were conducted utilizing the maximum-likelihood method within MEGA 7.0.26 software [46]. The 1,000 bootstrap method was employed to assess the reliability and statistical significance of the phylogenetic trees. Due to the unavailability of full-length viral gene sequences of some diarrhea-associated viruses, the consensus gene regions for all analyzed viruses were trimmed for phylogenetic analysis (Table S1).

## 3. Results

### 3.1. Identification of Diarrhea-Associated Viruses in the Stunted Pigs

**Figure 1:**
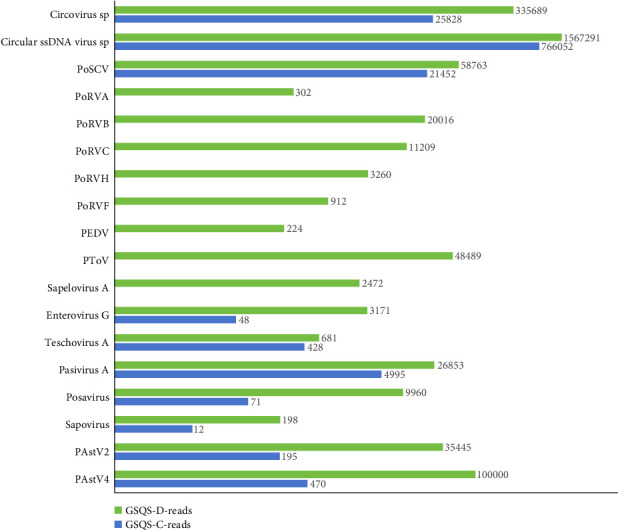
Species and number of reads annotated to viruses in the anal swab samples collected in 2022 from the Qingshan farm located in Gansu province of China were counted. Bars represent the number of reads for each virus. Green bars showed the viruses detected in the anal swab samples of stunted pigs, and blue bars represent the viruses detected in the clinical healthy pigs.

HTS of RNA and DNA libraries constructed with anal swab GSQS-D yielded a total of 16,715,303 raw sequence reads, with 2,224,633 reads annotating to mammalian viruses, and accounting for 14.9% of the total reads. The 18 mammalian viruses annotated in this manner were identified as belonging to 12 viral genera within seven viral families. All 15 RNA viruses within these were associated with porcine diarrhea, and consisted of five members of the *Sedoreoviridae* (PoRVA, PoRVB, PoRVC, PoRVH and PoRVF), five members of the *Picornaviridae* (enterovirus G, teschovirus, pasivirus A, posavirus, and sapelovirus A), one member of the *Caliciviridae* (sapovirus), and four members of the *Coronaviridae* (PEDV), *Tobaniviridae* (PToV), and *Astroviridae* (PAstV2 and PAstV4). Of note is that this is the first identification of RVF in pigs, which has hitherto been detected exclusively in birds. The most abundant viral reads were annotated to PAstV4, followed by PToV, PAstV2, pasivirus A, and PoRVB (Figure 1). Among the identified DNA viruses, Circular ssDNA virus *spp*. was the most abundant, followed by circovirus *spp*., and porprismacovirus (Figure 1).

In the anal swab GSQS-H, 819,551 reads aligned to 10 mammalian viruses (Figure 1). All were also present in stunted pigs, but in comparatively low abundance. Of note is that PoRVs, PEDV, torovirus, and sapelovirus A were present only in the stunted pigs (Figure 1). Further RT-PCR detection was performed to validate the viromic data and to determine the prevalence rates for viruses present exclusively in the stunted pigs. Results were as follows: 4.6% for PEDV, 9.1% for PoRVA, 18.2% for PoRVF, 40.9% for PoRVC, 54.6% for PoRVB, 54.6% for PoRVH, 59.1% for Sapelovirus A, and 59.1% for Torovirus. For other diarrhea-associated RNA viruses, the positive rates were 36.4% for posavirus, 22.7% for PAstV4, 59.1% for PAstV2, 59.1% for enterovirus G, 50.0% for teschovirus, and 95.5% for pasivirus. With the exception of GSQS13, 3–11 viruses were found in each sample from stunted pigs, with samples GSQS2 and GSQS21 containing the highest numbers (Table 2).

### 3.2. Complexity of PoRV Strains Circulated in the Sampled Farm

For the PoRVA-GSQS2022 strain, 22 contigs were obtained with lengths ranging from 240 to 735 bp. Their nucleotide (nt) identities with the most similar reference strains were 91.5%−97.8%. Two VP4 and one VP7 coding gene sequences were identified lengths of <350 bp. Sequence comparison showed that VP4-1 belonged to the P[13] genotype, being closely related to reference strain CHN/ZJ/XH3/2022/G9P[13] with an nt identity of 95.0%. VP4-2 belonged to genotype P[27], sharing the closest genetic relationship with reference strain CMP034 with an nt identity of 93.5%. VP7 belonged to genotype G4 and was most closely related to reference strain CHN/CY/DY/2022/G4P[6]I1, sharing an nt identity of 97.8%.

**Figure 2:**
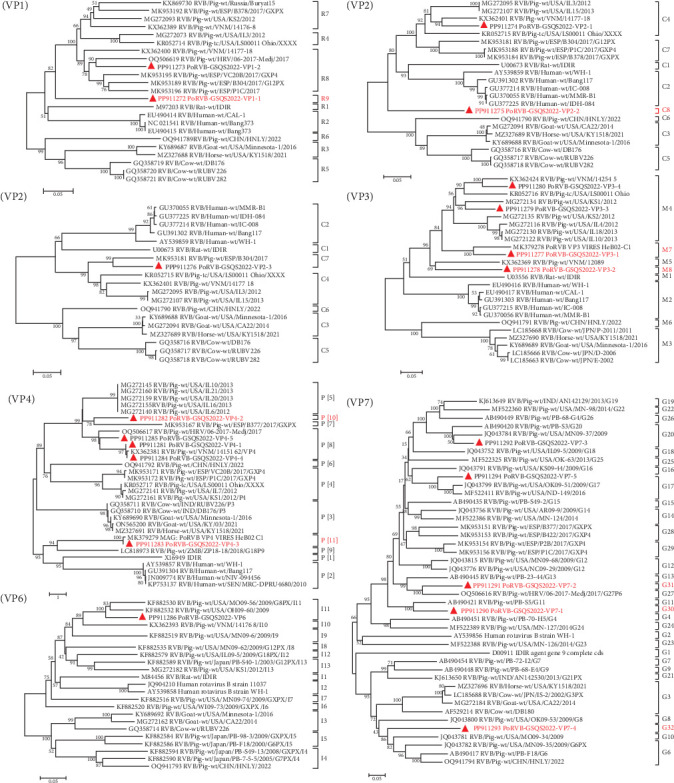
Phylogenetic trees based on the nucleotide sequences of VP1-VP4, VP6, VP7 gene segments of PoRVB-GSQS2022 strains. Neighbor-joining method with 1000 bootstrap replicates was used to construct the trees based on the nucleotide sequences of different gene segments of PoRVB-GSQS2022 and other reference strains. PoRVB-GSQS2022 strains identified in this study are identified with red triangles. Strains listed in red belong to new genotypes, with their identities with reference strains of known genotypes shown as below the genotyping cut-off values. Scale: evolutionary distances determined by the nucleotide substitution rate per site.

**Figure 3:**
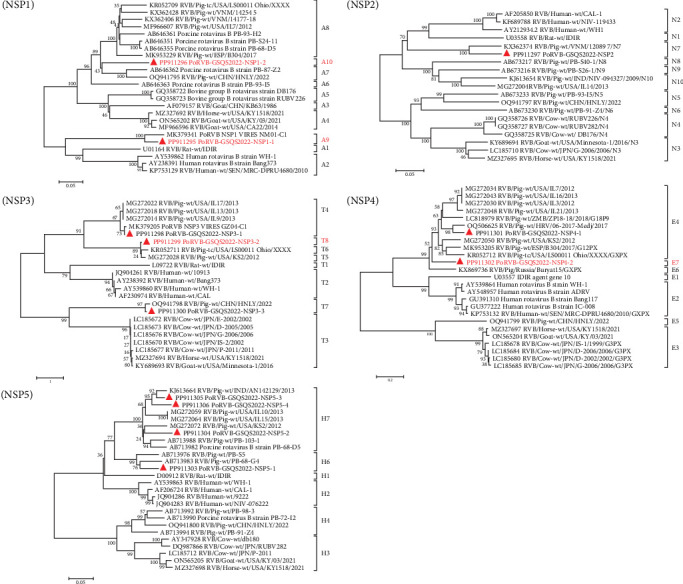
Phylogenetic trees based on the nucleotide sequences of NSP1-NSP5 gene segments of PoRVB-GSQS2022 strains. Neighbor-joining method with 1000 bootstrap replicates was used to construct the trees based on the nucleotide sequences of different gene segments of PoRVB-GSQS2022 and reference strains. PoRVB strains identified in this study are identified with red triangles. Strains listed in red belong to new genotypes, with their identities with reference strains of known genotypes shown as below the genotyping cut-off values.

A total of 131 contigs, ranging in length from 240 bp to 2,286 bp, were obtained for all gene segments of PoRVB-GSQS2022, sharing 72.0%−94.5% nt identities with the most closely related reference strains. Sequence comparisons revealed multiple variant gene sequences for individual PoRVB segments encoding VP1 (2), VP2 (3), VP3 (4), VP4 (5), VP7 (5), NSP1 (2), NSP3 (3), NSP4 (2), and NSP5 (4), and the most similar reference strain for each segment or variant of PoRVB was listed in Table 3. These have been deposited in GenBank under accession numbers PP911272-PP911306. According to the proposed genotype constellations of RVB strains, the nucleotide percent identity cut-off values were 80%, 80%, 81%, 78%, 79%, 77%, 76%, 83%, 78%, 76%, and 79% for gene segments VP7, VP4, VP6, VP1, VP2, VP3, NSP1, NSP2, NSP3, NSP4, and NSP5/6, respectively [47, 48], with one or more new genotypes being proposed for most segments of the PoRVB-GSQS strain. Among these, VP1-1 exhibited the closest genetic relationship to the RVB/Pig-wt/ESP/P1C/2017 (nt identity <78%), thereby defined as a new genotype R9, and VP1-2 was of genotype R8. Three fragments of the VP2 gene (>1200 bp) were obtained with VP2-1 and VP2-2 aligned to the 3′-terminal half of the reference genes, and VP2-3 aligned to the 5′-terminal half of the reference gene. Therefore, phylogenetic analysis based on these VP2 gene fragments was performed, and VP2-1 and VP2-3 were found to cluster with C4 and C7 genotype reference strains, respectively, while VP2-2 branched separately in the phylogenetic tree, belonging to a new genotype C8. Of the 4 contigs of VP3, VP3-2 shared a close relationship with the RVB/Pig-wt/VNM/12089 strain (nt identity <77%), creating a new genotype M8, while VP3-1 aligned within the M7 genotype and VP3-3, along with VP3-4, within the M4 genotype. For the outermost capsid protein VP4, VP4-2 formed a new genotype P[10], and VP4-3 was mostly related to the unknown genotype reference strain PoRVB_VP4_VIRES_HeB02_C1, defining another new genotype P[11]. The others (VP4-1, VP4-4, and VP4-5) clustered with the P[8] reference strains. The obtained 5 variant gene sequences of VP7 shared 57.7%−72.2% nt sequence identity, among which VP7-1, VP7-2, and VP7-4 were defined as 3 new genotypes, respectively (G30, G31, and G32), with the remaining two gene sequences belonging to genotypes G20 and G17, respectively (Figure 2). NSP1-1 shared 88.4% nt identity with the PoRVB_NSP1_VIRES_NM01_C1 strain of a new genotype A9 and NSP1-2 shared <76% nt sequence identity with the existing strain, representing a new genotype A10. NSP2 gene sequence clustered in the N7 genotype with RVB/Pig-wt/VNM/12089_7 strain. NSP3-2 was most closely related to the T6 genotype reference strains but not on the same branch, and has therefore been tentatively assigned as T8 genotype. NSP3-1 and NSP3-2 belonged to the T4 and T7 genotypes respectively. NSP4-1 shared the closest genetic relationship with genotype E4 reference strain, with NSP4-2 forming a separate clade in the evolutionary tree and representing a new genotype E7. For NSP5, NSP5-1 belonged to genotype H6, and the others (NSP5-2, NSP5-3, and NSP5-4) to genotype H7 (nt identity 79.0%−85.7%) (Figure 3).

**Figure 4:**
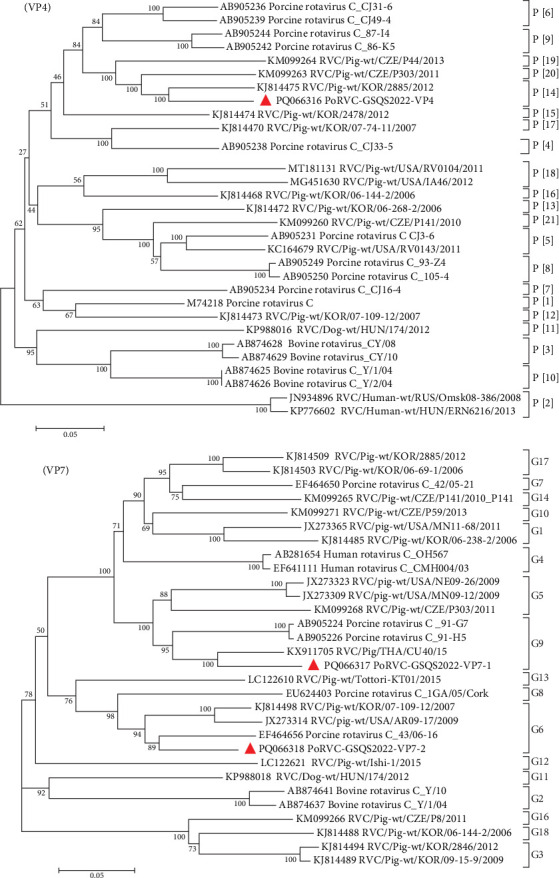
Phylogenetic trees based on the nucleotide sequences of VP4 and VP7 gene segments of PoRVC-GSQS2022 strains. Neighbor-joining method with 1000 bootstrap replicates was used to construct the trees based on the nucleotide sequences of different gene segments of PoRVC-GSQS2022 and other reference strains. The PoRVC-GSQS2022 strain identified in this study is identified by a red triangle.

A total of 41 contigs were obtained for PoRVC, ranging from 300 to 2,199 bp in length, and sharing 86.9%–96.1% nt identity with reference strains. The VP4 and two VP7 gene sequences were deposited in GenBank under accession numbers PQ066316-PQ066318. Genotyping based on the VP4 gene segment showed that the PoRVC-GSQS2022 strain clustered in the same branch as P[14] genotype reference strain RVC/Pig-wt/KOR/2885/2012 (nt identity, 88.3%). Of the two VP7 gene sequences, VP7-1 (996 bp) aligned with the G9 genotype, sharing a close relationship with the RVC/Pig/THA/CU40/15 strain (nt identity, 90.8%), while VP7-2 aligned with the G6 genotype, sharing the closest genetic relationship with the 43/06-16 strain (nt identity, 89.3%) (Figure 4).

**Figure 5:**
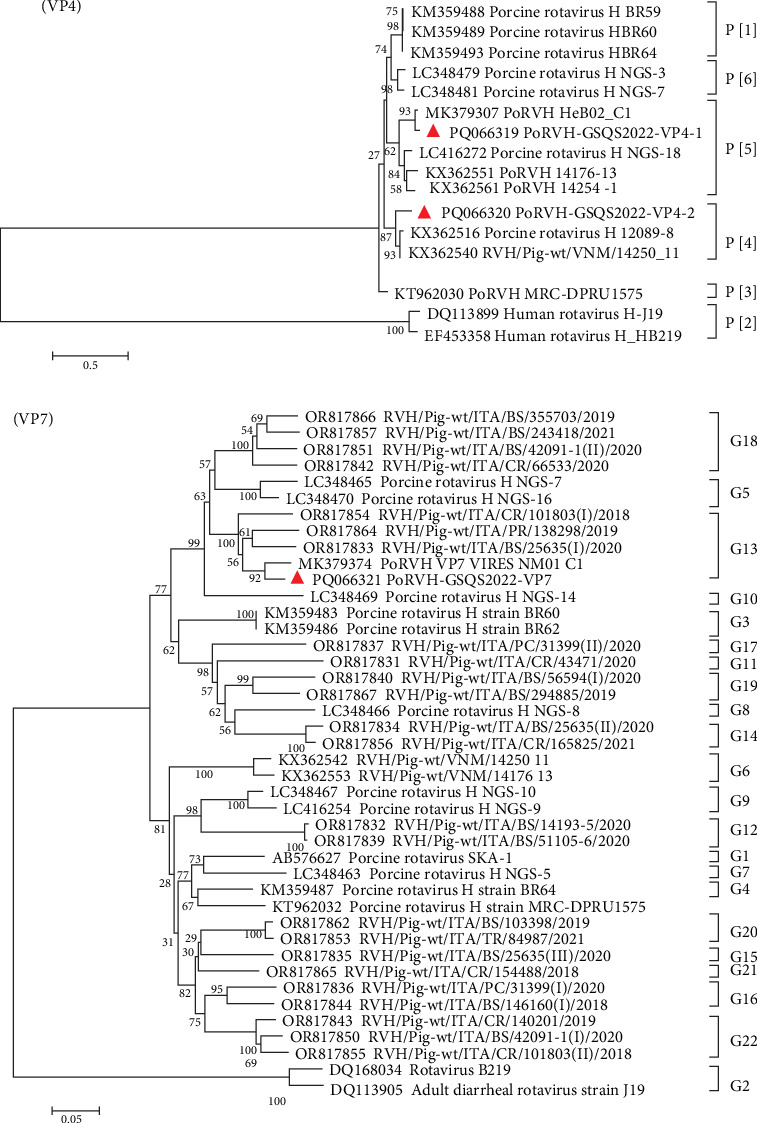
Phylogenetic trees based on the nucleotide sequences of VP4 and VP7 gene segments of PoRVH-GSQS2022 strains. Neighbor-joining method with 1000 bootstrap replicates was used to construct the trees based on the nucleotide sequences of different gene segments of PoRVH-GSQS2022 and reference strains. The PoRVH-GSQS2022 strain identified in this study is identified by a red triangle.

Forty-one contigs were obtained for PoRVH, ranging from 300 to 2823 bp in length. The nt identities between the PoRVH-GSQS2022 strain and the most similar reference strains ranged from 84.4% to 96.8%. Two variant VP4- and one VP7-coding sequences were identified and have been deposited in GenBank under accession numbers PQ066319-PQ066321. Phylogenetic analysis showed that VP4-2 clustered together with P[4] genotype reference strains, sharing 90.6% nt sequence identity with the most closely related reference strain RVH/Pig-wt/VNM/14250_11. VP4-1 aligned to genotype P[5] and was closely related to PoRVH_VP4_VIRES_HeB02_C1 strain (nt identity of 96.2%). In the phylogenetic tree constructed with VP7 gene sequences, PoRVH-GSQS2022 clustered in the same branch as the PoRVH_VP7_VIRES_NM01_C1 strain, with an nt identity of 94.7%, thereby forming genotype G13 (Figure 5).

**Figure 6:**
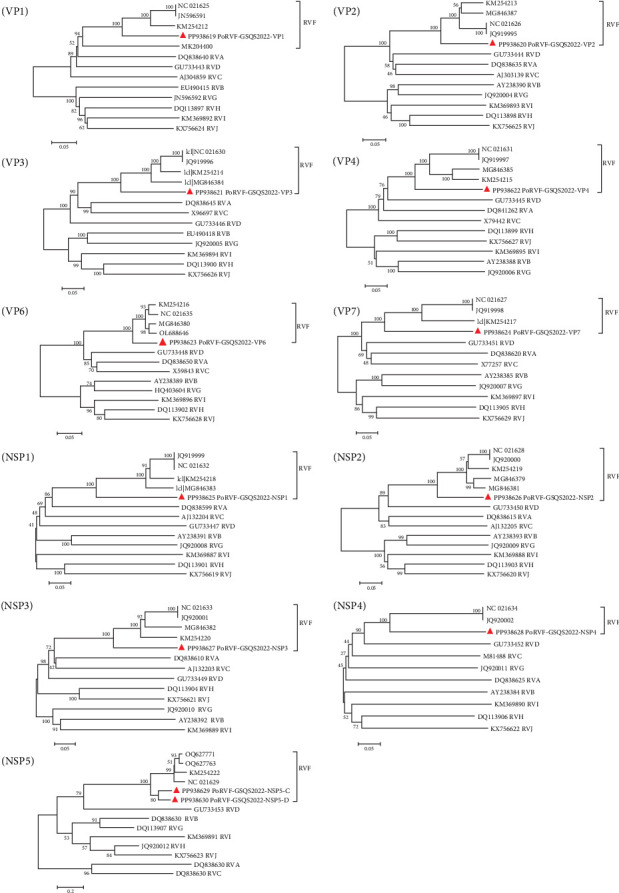
Phylogenetic trees based on the nucleotide sequences of 11 gene segments of PoRVF-GSQS2022 strains. Neighbor-joining method with 1000 bootstrap replicates was used to construct the trees based on the nucleotide sequences of different gene segments of PoRVF-GSQS2022 and reference strains of other rotavirus species. The PoRVF-GSQS2022 strain identified in this study is identified by a red triangle.

Twelve contigs of PoRVF were obtained through bioinformatics analysis of viral metagenomic data, corresponding to 11 gene segments of the PoRVF genome with two variant sequences of the NSP5 gene segment (NSP5-C and NSP5-D), and validated by RT-PCR and Sanger sequencing. Complete ORFs were obtained for the VP6, NSP3, and NSP5 gene segments, and nearly complete coding regions were obtained for the remaining gene segments, all of which have been deposited in GenBank under accession numbers PP938619-PP938630. Although both 3′ and 5′ RACE amplification techniques were used, all attempts to obtain the missing genomic sequences failed. Sequence comparison indicated that the nt sequence identities of the individual segments of the PoRVF strain first identified here (PoRVF-GSQS2022) and RVF reference strain 03V0568 ranged from 56.5% (NSP5) to 79.4% (VP2), while the aa sequence identities ranged from 46.2% (VP7) to 92.0% (VP2). Notably, PoRVF-GSQS2022 proteins VP4, NSP1, and NSP5 shared <60% aa sequence identity with reference strains, and <50% aa identity with VP7 and NSP4 (Table 4). The nt and aa identities between NSP5-C and NSP5-D coding genes were 83.9% and 86.5%, respectively. Further comparison of PoRVF-GSQS2022 with reference strains of all RV species indicated that PoRVF-GSQS2022 shared much lower sequence identities with other RV species than with RVF reference strains (Table 5), indicating that no reassortant events had occurred between PoRVF-GSQS2022 and strains of other RV species. Phylogenetic analysis based on the nt sequences of 11 gene segments from RV A–J species showed that PoRVF-GSQS2022 clustered in the same clade as the avian RVF reference strains, but branched separately from the RVF strains in all phylogenetic trees except for the VP1-constructed tree (Figure 6). The PoRVF strain obtained in this study was therefore significantly different from existing RVF reference strains, indicating that it is a new variant RVF strain.

### 3.3. Genetic Diversity of PAstVs and Other Diarrheal Viruses in the Stunted Pigs

**Figure 7:**
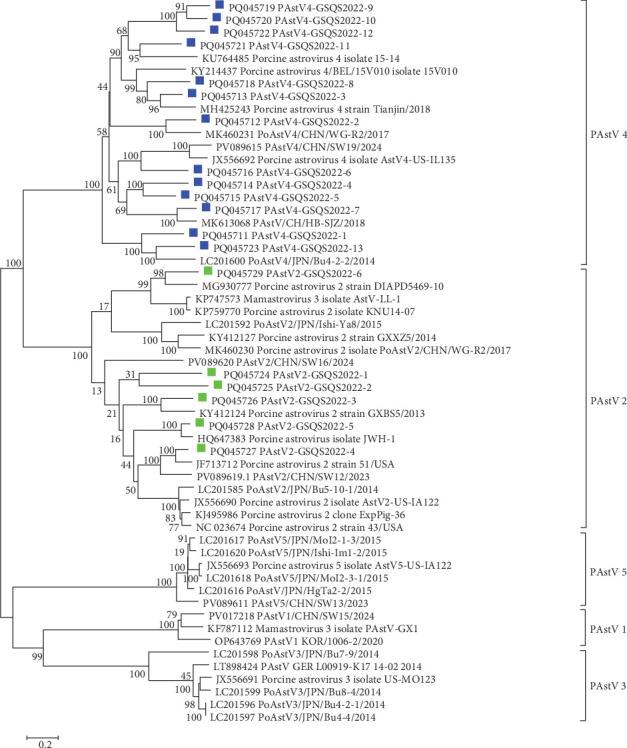
Phylogenetic tree based on the ORF2 coding sequence of astroviruses. The tree was constructed based on 19 PAstV ORF2 gene sequences and 38 reference PAstV1-PAstV5 ORF2 gene sequences. Phylogenetic analysis was conducted utilizing the neighbor-joining method within the Phylogeny module of DNASTAR with 1000 bootstrap replicates. The PAstV4-GSQS2022 and PAstV2-GSQS2022 strains are identified by blue and green squares, respectively.

A total of 375 contigs of PAstVs were obtained, including 19 ORF2 gene sequences of lengths >2000 bp, and sharing 40.3%−76.1% nt sequence identities. A phylogenetic tree constructed with 19 PAstVs and 38 reference ORF2 gene sequences showed that 6 PAstV2 variant strains (PAstV2-1 to PAstV2-6) clustered in the PAstV2 clade with the reference strains, sharing 48.5% to 83.6% nt identities. Thirteen PAstV4 variant strains (PAstV4-1 to PAstV4-13) detected in this study clustered within the PAstV4 clade, sharing 52.6%−78.9% nt sequence identities (Figure 7). Further sequence comparisons showed that the 6 PAstV2-GSQS2022 strains identified here shared 57.1%−65.4% nt and 51.0–65.0% aa sequence identities, and that the 13 PAstV4-GSQS2022 strains shared 36.9%−73.0% nt and 43.9%−78.2% aa sequence identities. Altogether, the PAstV-GSQS2022 strains identified in the stunted pigs exhibited a high degree of genetic diversity.

In addition to PoRVs and PAstVs, multiple diarrhea-associated viruses, including PEDV, torovirus, pasivirus A, and sapovirus, were also detected in the stunted pigs. PEDV was identified in only 1 sample (named PEDV-GSQS2022) and found to belong to genotype G2b through phylogenetic analysis with the nt sequence of the full-length S gene, being closely related to the South Korean strain K15CN5 collected in 2015 (nt identity, 99.4%). A torovirus, now classified as a member of the family *Tobaniviridae* (but formerly of the *Coronaviridae*), was detected in 59.1% of samples, and the complete genome sequence (28,302 nt) of the torovirus strain GSQS2022 was obtained, revealing a close relationship with the Chinese strain ZJU with an nt identity of 95.9%.

Multiple picornaviruses were found in the anal swabs, with pasivirus A as the most prevalent, with an infection rate of 95.5%. Sequence analysis of a nearly complete genome (6908 nt) of pasivirus A strain GSQS2022 revealed its highest identity with reference strain SPaV-A/GER/L01061-K07_15-03/2015 (nt 84.2% and aa 93.7%). A nearly complete genome sequence (8,591 nt) of posavirus strain GSQS2022 was generated and found to be most closely related to the United States strain 8805 with nt and aa identities of 86.9% and 96.9%, respectively. The length of the porcine teschovirus (PTV) A genome is about 7200, and 5583 nt of strain PTV-GSQS-2022 was obtained in this study. It belonged to genotype A3 and was most similar to the Chinese strain PTV-China/SWU-E5/2018 in 2018, with nt and aa identities of 87.5% and 95.3%, respectively. For porcine sapelovirus (PSV) A, a nearly complete genomic sequence (6978 nt) was obtained, which shared the closest relationship with the HuN2 strain identified in China in 2015, with shared nt and aa identities of 88.8% and 96.5%, respectively. In addition, a total of 11 contigs ranging from 351 bp to 768 bp were annotated to sapovirus. Sequence comparisons showed that two sapoviral genotypes (GVI and GXI) were identified in the samples from the Gansu farm.

## 4. Discussion

Stunted pigs were commonly present in the industrialized pig farms, especially for those with poor bio-security management, the occurrence and development of which was associated with multiple factors [49], but most of them were caused by infection of microorganisms, particularly enteric viruses. After infection, viruses such as RVs can cause varying degrees of villous damage in infected piglets, resulting in impaired nutrient absorption and incomplete digestion [25, 50, 51]. However, virus detection in the stunted pigs was rarely performed with traditional methods or HTS because of the low economic value. In this study, an HTS-based viral metagenomics approach was utilized to explore the potential causes of diarrhea and growth retardation in stunted pigs from a farm in Gansu province. As a result, 18 different mammalian viruses within seven viral families were identified in anal swabs, indicating a complex and diverse viral community in the sampled farm. Among these, only the RNA viruses were associated with diarrhea, with at least three viruses detected in each sample. Notably, one sample provided evidence for a mixed infection with 10 different viruses, indicating a high co-infection rate with porcine diarrhea viruses and a diverse viral community in the sampled farm. In many large-scale pig operations, stunted, sick, or disabled pigs raised in the nursery house are not transferred to the fattening house if their body weight at 3 months is <10 kg. Instead, they are generally gathered in separate housing rather than being killed, since the pig breeder's profits are tightly related to the survival rate of the pigs. The viromic results obtained here suggest that stunted pigs may serve as a hotbed for the propagation of diarrhea-associated viruses and contribute to the generation of new viruses or variants. Growth-retarded pigs should therefore be treated as early as possible.

While PEDV and PoRVA are the primary pathogens responsible for causing diarrhea in piglets, leading to significant losses in the pig industry [52, 53], they received only a small number of reads in the samples from stunted pigs, with only 1 sample testing positive for PEDV and 2 for PoRVA. It appears, therefore, that these two diarrheal viruses are not the primary causes of stunting in this farm. RVB has been reported to exhibit more genetic diversity than members of other RV species, with porcine strains suggested as being ancestors of those infecting other species [48, 54]. Among the 5 PoRVs identified in this study, PoRVB had the most abundant contigs, and multiple variant gene sequences were detected for each gene segment. Except for the gene segments encoding VP6 and NSP2, all others contained two or more genotypes. In 8 of 11 gene segments, new variant gene sequences were found, differing significantly from existing reference sequences and further confirming the genetic diversity of RVB strains. Given the limited numbers of anal swab samples tested, it is not possible to determine the derivative strains of the various 11 gene segments; however, it is apparent that multiple variant strains of PoRVB were circulating in the sampled farm. Previous studies have shown that RV co-infection leads to the emergence of new variant strains [31, 55], and therefore we propose that the emergence of numerous new genotypes of PoRVB strains may be attributed to the complex mixed infection of multiple variants in the same host.

RVF was originally identified in chickens in 1984 and is distinguished from other RVs by their unique electrophoretic migration profiles 4–1–2–2–2 and specific antisera [56]. RVF has been continuously found in turkeys, broiler chickens, and wild birds, but is exclusively found in avians [32, 57–61]. So far, only one complete genome sequence of RVF (03V0568) has been recorded in GenBank. The strain was derived from chicks with diarrhea and growth retardation symptoms on farms in northern Germany [62]. However, RVF in broiler chickens and birds reported in Brazil was detected in asymptomatic flock samples [57, 58, 61]. Overall, the knowledge of the epidemiology and clinical manifestation of RVF is still limited. According to previous reports, the International Committee on Taxonomy of Viruses (ICTV) classified RVF as the avian-specific RV species. In our study, RVF was unexpectedly found in growth-retarded pigs. Similar to the German RVF strain 03V0568, the sampled herds also showed growth retardation and mild diarrhea symptoms. However, a variety of porcine diarrhea-associated viruses other than RVF were found in the collected samples through metagenomic analysis, thus, the association between RVF and swine clinical diseases cannot be definitively established. The RVF strain PoRVF-GSQS2022 identified here exhibits significant differences in gene sequence homology and phylogenetic relationships compared to existing RVF reference strains, indicating that it represents a novel variant of RVF. Due to low gene sequence homology with known reference RVF strains, we were unable to identify the original host. The identification of this strain in pig samples suggests the potential for cross-species transmission of RVF. Notably, two NSP5 variants were identified in the PoRVF-GSQS2022 genome, which were specifically localized to diarrhea and nondiarrhea pigs through targeted PCR. This observation raises the hypothesis that differing host environments and viral species in these two groups may induce distinct alterations in the host fitness of RVF viruses. In summary, our study contributes to the genomic data of RVF and broadens the spectrum of known infected hosts. Further exploration on RVF prevalence is required to enhance our understanding of the evolution and potential of viral spillover.

## 5. Conclusion

The present study characterized multiple diarrheal-associated viruses in stunted pigs. PoRVBs showed genetic diversity, with multiple new genotypes proposed. RVF was first detected in pigs, sharing low identities with previously reported avian strains.

## Figures and Tables

**Table 1:** PCR validation primers for diarrhea-associated viruses.

Primer	Primer sequence 5′→3′	Product size
PEDV-F	TAAARCAYTTYTTCTTYGCACAG	1000 bp
PEDV-R	CCATCATCAGADARAATCATCATWGA	

PoRVA-F	ATGGAGGTTCTGTACTCATTGT	130 bp
PoRVA-R	CTTCTAATGGAAGCTACTGTGA	

PoRVB-F	GCAGGAAATGGTCTCACACAGT	510 bp
PoRVB-R	GCATCTTGGGCGTTATCTACTC	

PoRVC-F	ATGGCAACACATTTCATACTGG	330 bp
PoRVC-R	ATTCATAAACTGGATTTTCACG	

PoRVH-F	CGGTGGTGCTTTTGATAATAA	249 bp
PoRVH-R	GCAAACAAACAAGCAAATAAC	

PoRVF-F	ACTGAGAAGGGAATGGTTTAT	331 bp
PoRVF-R	TTGCCACTTCAGACATCACTA	

Enterovirus-F	TGGAGGYSAKGGTGTDGTYG	858 bp
Enterovirus-R	ARTGRTCRCTGTCTGGRGGR	

Teschovirus-F	ATGGGCACAATACATTCAGT	495 bp
Teschovirus-R	GCTTAGGACCTGGAGAACA	

Sapelovirus-F	CATCCCAAAGTCAAAACATCT	255 bp
Sapelovirus-R	GGTTAGAAATGCCAACAAAAG	

Pasivirus-F	GGDGAGTCCATGGATCTTTGT	889 bp
Pasivirus-R	CAYCKWGTAAGTCCTTGTTCAA	

Posavirus-F	TACCTTGCTCTGACCGATTAC	260 bp
Posavirus-R	TGGTATGACAAAGAACGGACT	

Sapovirus-F	TCAGTTGAGTTCCGCATTAGC	500 bp
Sapovirus-R	CTGCCAGACTACCTGAAGTGCTA	

Torovirus-F	AAGGAACAAAAGAGCCAGGA	1030 bp
Torovirus-R	ATAGGATGCCGTTAGGTCGT	

Astrovirus 2-F	GTAACCCTCCTACAGGTATTTG	480 bp
Astrovirus 2-R	TAATGCCTTTACAGGTGGTCTA	

Astrovirus 4-F	ATTCACACTTTGGGGAAGACTG	500 bp
Astrovirus 4-R	TTCTTTTTCTTGTTGGCGATTC	

**Table 2:** The PCR detection results of diarrhea-associated viruses.

Virus Sample	pasivirus	EV-G	PTV-A	PSV	PToV	PAstV2	PoRVB	PoRVH	PoRVC	posavirus	PAstV4	PoRVF	SaV	PoRVA	PEDV	Number of co-infected viruses
GSQS2	+	+	+	+	+	+	+	+	+	+	—	—	+	—	—	11
GSQS21	+	+	+	+	+	—	+	+	+	+	—	+	—	—	—	10
GSQS7	+	+	—	+	+	—	+	+	+	+	—	—	—	+	—	9
GSQS18	+	—	+	—	+	+	+	+	+	+	+	+	—	—	—	9
GSQS16	+	—	—	+	+	+	+	—	+	+	—	+	—	—	—	8
GSQS5	+	+	+	+	—	—	+	—	—	—	—	—	+	+	—	7
GSQS6	+	+	—	+	+	—	+	+	—	—	+	—	—	—	—	7
GSQS9	+	+	—	+	+	+	+	—	—	—	—	—	+	—	—	7
GSQS12	—	—	—	—	+	—	+	+	+	—	—	+	—	—	+	7
GSQS14	+	+	+	—	+	+	—	—	+	+	—	—	—	—	—	7
GSQS20	+	+	+	+	+	+	—	+	—	—	+	—	—	—	—	7
GSQS11	+	+	+	+	—	+	—	+	—	—	—	—	—	—	—	6
GSQS15	+	+	+	+	+	+	—	—	—	—	—	—	—	—	—	6
GSQS22	+	+	+	+	—	+	—	—	—	+	—	—	—	—	—	6
GSQS3	+	+	—	+	+	+	—	—	—	—	—	—	—	—	—	5
GSQS8	+	—	+	—	—	—	+	+	+	—	—	—	—	—	—	5
GSQS17	+	—	—	—	+	+	+	+	—	—	+	—	—	—	—	5
GSQS1	+	+	—	—	—	+	—	+	—	—	—	—	—	—	—	4
GSQS4	+	—	—	+	—	+	—	—	+	—	—	—	—	—	—	4
GSQS19	+	—	+	—	—	—	+	—	—	—	+	—	—	—	—	4
GSQS10	+	—	—	—	—	—	—	+	—	+	—	—	—	—	—	3
GSQS13	—	—	—	—	—	—	—	—	—	—	—	—	—	—	—	0

**Table 3:** Gene segment analysis of PoRVB-GSQS2022.

Gene segment	Length (nt)	Information of most similar strain		nt homology	Genotype
VP1-1	1989	MK953196	RVB/Pig-wt/ESP/P1C/2017	74.1%	**R9**
VP1-2	2169	OQ506619	RVB/Pig-wt/HRV/06-2017-Medj/2017	84.1%	R8

VP2-1	1305	KX362401	RVB/Pig-wt/VNM/14177-18	87.0%	C4
VP2-2	1218	KR052715	RVB/Pig-tc/USA/LS00011-Ohio/XXXX	76.3%	**C8**
VP2-3	1233	MK953181	RVB/Pig-wt/ESP/B304/2017	83.0%	C7

VP3-1	1851	MK379278	PoRVB-VP3-VIRES-HeB02-C1	85.4%	**M7**
VP3-2	2283	KX362369	RVB/Pig-wt/VNM/12089	75.2%	**M8**
VP3-3	2286	MG272134	RVB/Pig-wt/USA/KS1/2012	83.1%	M4
VP3-4	1218	KX362424	RVB/Pig-wt/VNM/14254-5	86.6%	M4

VP4-1	2061	OQ506617	RVB/Pig-wt/HRV/06-2017-Medj/2017	82.0%	P[8]
VP4-2	1188	MG272140	RVB/Pig-wt/USA/IL6/2012	78.2%	**P[10]**
VP4-3	1023	MK379279	PoRVB-VP4-VIRES-HeB02-C1	93.8%	**P[11]**
VP4-4	1068	KX362381	RVB/Pig-wt/VNM/14151-62	87.4%	P8
VP4-5	996	KX362381	RVB/Pig-wt/VNM/14151-62	82.7%	P8

VP6-1	858	KF882532	RVB/Pig-wt/USA/OH09-60/2009	87.6%	I11

VP7-1	612	MF522386	RVB/Pig-wt/USA/MN-124/2014	74.1%	**G30**
VP7-2	621	AB490445	PoRVB-strain: PB-23-44/2005	76.8%	**G31**
VP7-3	681	JQ043784	RVB/Pig-wt/USA/MN09-37/2009	87.5%	G20
VP7-4	684	JQ043781	RVB/Pig-wt/USA/MO09-34/2009	74.4%	**G32**
VP7-5	681	MF522411	RVB/Pig-wt/USA/ND-149/2016	87.6%	G17

NSP1-1	627	MK379341	PoRVB-NSP1-VIRES-NM01-C1	88.4%	**A9**
NSP1-2	960	AB646361	RVB/Pig-wt/Japan/PB-93-H2	72.0%	**A10**

NSP2-1	852	KX362374	RVB/Pig-wt/VNM/12089-7	89.3%	N7

NSP3-1	804	MK379205	PoRVB-NSP3-VIRES-GZ04-C1	94.5%	T4
NSP3-2	765	KR052711	RVB/Pig-tc/USA/LS00011-Ohio/XXXX	79.7%	**T8**
NSP3-3	783	OQ941798	RVB/Pig-wt/CHN/HNLY/2022	86.5%	T7

NSP4-1	663	OQ506625	RVB/Pig-wt/HRV/06-2017-Medj/2017	89.0%	E4
NSP4-2	627	MK953205	RVB/Pig-wt/ESP/B304/2017/G12PX	75.9%	**E7**

NSP5-1	519	AB713976	PoRVB-strain: PB-S5	85.4%	H6
NSP5-2	522	AB713982	PoRVB-strain: PB-68-D5	84.2%	H7
NSP5-3	522	KJ613664	RVB/Pig-wt/IND/AN142129/2013	93.3%	H7
NSP5-4	510	AB713988	PoRVB-strain: PB-103-1	81.2%	H7

*Note*: The new RVB genotypes identified in this study were marked in bold.

**Table 4:** Homology analysis of 11 putative open reading frames of RVF.

Gene segment	Length (aa | nt)	Most similar Strain		Length (aa | nt)	aa homology	nt homology
VP1 (partial 77.3%)	840|2520	YP_008145313	Chicken/03V0568/DEU/2003/Germany	1086|3296	86.3%	77.6%
VP2 (partial 91.6%)	828|2484	YP_008145314	Chicken/03V0568/DEU/2003/Germany	904|2769	92.0%	79.4%
VP3 (nearly complete 99.7%)	692|2082	YP_008145318	Chicken/03V0568/DEU/2003/Germany	694|2174	71.8%	70.9%
VP4 (nearly complete 99.5%)	734|2202	YP_008145319	Chicken/03V0568/DEU/2003/Germany	738|2246	55.4%	64.0%
**VP6 (complete 100.0%)**	396|1188	YP_008145323	Chicken/03V0568/DEU/2003/Germany	396|1314	82.8%	75.8%
VP7 (partial 96.3%)	284|884	YP_008145315	Chicken/03V0568/DEU/2003/Germany	295|990	46.2%	60.4%
NSP1 (partial 71.8%)	393|1179	YP_008145320	chicken/03V0568/DEU/2003/Germany	547|1791	59.3%	64.1%
NSP2 (partial 91.5%)	291|873	YP_008145316	Chicken/03V0568/DEU/2003/Germany	318|1068	75.9%	75.1%
**NSP3 (complete 100.0%)**	370|1249	YP_008145321	Chicken/03V0568/DEU/2003/Germany	370|1309	74.9%	71.5%
NSP4 (partial 83.4%)	141|411	YP_008145322	Chicken/03V0568/DEU/2003/Germany	169|678	46.8%	56.5%
**NSP5-D (complete 100.0%)**	219|677	YP_008145317	Chicken/03V0568/DEU/2003/Germany	218|706	59.0%	66.2%
**NSP5-C (complete 100.0%)**	219|676	YP_008145317	Chicken/03V0568/DEU/2003/Germany	218|706	59.0%	66.6%

*Note*: The gene segments of PoRVF strain with full-length sequences obtained were marked in bold.

**Table 5:** Comparison of nt homology between GSQS2022 and different RV species.

	RVA	RVB	RVC	RVD	RVF	RVG	RVH	RVI	RVJ
VP1	DQ838640 (60.4%)	EU490415 (39.6%)	AJ304859 (57.7%)	GU733443 (60.8%)	JN596591 (78.5%)	JN596592 (42.1%)	DQ113897 (40.5%)	KM369892 (41.2%)	KX756624 (40.3%)
VP2	DQ838635 (59.6%)	AY238390 (33.2%)	AJ303139 (58.0%)	GU733444 (58.0%)	JQ919995 (80.1%)	JQ920004 (36.5%)	DQ113898 (36.9%)	KM369893 (35.1%)	KX756625 (34.0%)
VP3	DQ838645 (53.0%)	EU490418 (31.1%)	X96697 (49.2%)	GU733446 (52.9%)	JQ919996 (73.0%)	JQ920005 (33.5%)	DQ113900 (32.1%)	KM369894 (34.3%)	KX756626 (34.2%)
VP4	DQ841262 (51.9%)	AY238388 (31.9%)	X79442 (49.1%)	GU733445 (50.3%)	JQ919997 (68.2%)	JQ920006 (32.3%)	DQ113899 (34.5%)	KM369895 (31.6%)	KX756627 (32.7%)
VP6	DQ838650 (50.2%)	AY238389 (34.2%)	X59843 (49.4%)	GU733448 (52.6%)	HQ403603 (76.8%)	HQ403604 (35.2%)	DQ113902 (35.4%)	KM369896 (35.4%)	KX756628 (33.7%)
VP7	DQ838620 (42.4%)	AY238385 (33.1%)	X77257 (48.1%)	GU733451 (52.3%)	JQ919998 (61.9%)	JQ920007 (33.3%)	DQ113905 (34.0%)	KM369897 (31.1%)	KX756629 (33.6%)
NSP1	DQ838599 (34.2%)	AY238391 (41.6%)	AJ132204 (36.0%)	GU733447 (30.1%)	JQ919999 (67.5%)	JQ920008 (41.4%)	DQ113901 (41.5%)	KM369887 (35.8%)	KX756619 (36.5%)
NSP2	DQ838615 (50.6%)	AY238393 (31.9%)	AJ132205 (49.2%)	GU733450 (57.2%)	JQ920000 (76.3%)	JQ920009 (31.1%)	DQ113903 (37.3%)	KM369888 (33.1%)	KX756620 (33.4%)
NSP3	DQ838610 (39.0%)	AY238392 (38.8%)	AJ132203 (37.1%)	GU733449 (36.1%)	JQ920001 (72.1%)	JQ920010 (41.1%)	DQ113904 (44.4%)	KM369889 (34.4%)	KX756621 (44.0%)
NSP4	DQ838625 (35.1%)	AY238384 (34.0%)	X83967 (42.4%)	GU733452 (35.1%)	JQ920002 (60.0%)	JQ920011 (47.8%)	DQ113906 (31.3%)	KM369890 (39.3%)	KX756622 (32.9%)
NSP5-D	DQ838630 (34.7%)	AY238394 (33.5%)	M81488 (32.3%)	GU733453 (37.6%)	JQ920003 (65.8%)	JQ920012 (34.8%)	DQ113907 (32.0%)	KM369891 (30.2%)	KX756623 (33.2%)
NSP5-C	DQ838630 (35.4%)	AY238394 (33.2%)	M81488 (30.5%)	GU733453 (38.1%)	JQ920003 (66.2%)	JQ920012 (34.7%)	DQ113907 (33.5%)	KM369891 (31.2%)	KX756623 (33.9%)

## Data Availability

The datasets supporting the conclusions of this article are included within the article and its additional file.
