# Supplementary material for: Complexity of Diarrhea-Associated Viruses in Stunted Pigs Identified by Viral Metagenomics
Source: Transbound Emerg Dis. 2025 Jun 18;2025:1974716. doi: 10.1155/tbed/1974716 (PMC12197562; doi:10.1155/tbed/1974716)
Supplement: Supporting Information — Supporting Table S1 [file 1974716.f1.docx]

| Table S1 Supplementary information for phylogenetic analysis | | | | |
| --- | --- | --- | --- | --- |
| Virus species | Sequence to be analyzed | Reference sequence | gene region | |
|  |  |  | Starting  position | Ending  position |
| PoRVB | VP1 | PP669286 | 1450 | 2177 |
|  | VP2 (VP2-1/VP2-2) | MK953188 | 1505 | 2371 |
|  | VP2 (VP2-3) | MK953181 | 1 | 1232 |
|  | VP3 | PP669288 | 1072 | 1875 |
|  | VP4 | MK953172 | 1250 | 2248 |
|  | VP6 | PP669285 | 1 | 858 |
|  | VP7 | MK953154 | 42 | 643 |
|  | NSP1 | PP669289 | 546 | 1174 |
|  | NSP2 | PP669290 | 106 | 956 |
|  | NSP3 | OQ506624 | 103 | 784 |
|  | NSP4 | MK953212 | 28 | 641 |
|  | NSP5 | OQ506626 | 13 | 524 |
| PoRVC | VP4 | KM099264 | 196 | 2235 |
|  | VP7 | KM099271 | 13 | 1023 |
| PoRVH | VP4 | KX362561 | 16 | 609 |
|  | VP7 | OR817860 | 22 | 741 |
| PoRVF | VP1 | NC_021625 | 757 | 3276 |
|  | VP2 | NC_021626 | 245 | 2728 |
|  | VP3 | NC_021630 | 34 | 2115 |
|  | VP4 | NC_021631 | 21 | 2251 |
|  | VP6 | NC_021635 | 26 | 1213 |
|  | VP7 | NC_021627 | 45 | 936 |
|  | NSP1 | NC_021632 | 27 | 1205 |
|  | NSP2 | NC_021628 | 137 | 1009 |
|  | NSP3 | NC_021633 | 21 | 1280 |
|  | NSP4 | NC_021634 | 115 | 528 |
|  | NSP5 | NC_021629 | 16 | 701 |
| PAstV | ORF2 | KF787112 | 4624 | 6619 |
